# Integrated Analysis of lncRNA–mRNA Regulatory Networks Related to Lipid Metabolism in High-Oleic-Acid Rapeseed

**DOI:** 10.3390/ijms24076277

**Published:** 2023-03-27

**Authors:** Xiaodan Wang, Dongfang Zhao, Xi Li, Bingqian Zhou, Tao Chang, Bo Hong, Chunyun Guan, Mei Guan

**Affiliations:** College of Agriculture, Hunan Agricultural University, Hunan Branch of National Oilseed Crops Improvement Center, Southern Regional Collaborative Innovation Center for Grain and Oil Crops in China, Changsha 410128, China

**Keywords:** high-oleic-acid rapeseed, lncRNA–mRNA, lipid metabolism, seed development

## Abstract

A high oleic acid content is considered an essential characteristic in the breeding of high-quality rapeseed in China. Long-chain non-coding RNA (lncRNA) molecules play an important role in the plant’s growth and its response to stress. To better understand the role of lncRNAs in regulating plant reproductive development, we analyzed whole-transcriptome and physiological data to characterize the dynamic changes in lncRNA expression during the four representative times of seed development of high- and low-oleic-acid rapeseed in three regions. We identified 21 and 14 lncRNA and mRNA modules, respectively. These modules were divided into three types related to region, development stages, and material. Next, we analyzed the key modules related to the oil content and the oleic acid, linoleic acid, and linolenic acid contents with physiological data and constructed the key functional network analysis on this basis. Genes related to lipid metabolism, such as *3-ketoacyl-CoA synthase 16* (*KCS16*) and *acyl-CoA:diacylglycerol acyltransferase 1* (*DGAT1*), were present in the co-expression network, suggesting that the effect of these genes on lipid metabolism might be embodied by the expression of these lncRNAs. Our results provide a fresh insight into region-, development-stage-, and material-biased changes in lncRNA expression in the seeds of *Brassica napus*. Some of these lncRNAs may participate in the regulatory network of lipid accumulation and metabolism, together with regulated genes. These results may help elucidate the regulatory system of lncRNAs in the lipid metabolism of high-oleic-acid rapeseed seeds.

## 1. Introduction

Oilseed rape (*Brassica napus* L.) is one of the four largest oilseed crops in the world and the largest oilseed crop in China [1]. With the improvement in living standards, the population’s demand for high-quality rapeseed oil has also increased. Canola seeds predominantly contain five fatty acids: palmitic acid (16:0), stearic acid (18:0), oleic acid (18:1), linoleic acid (18:2), and linolenic acid (18:3) [2]. Among them, oleic acid plays an important role in human health as an essential fatty acid. For example, it aids in digestion and absorption, can reduce the content of cholesterol in the blood, softens blood vessels, prevents the formation of thrombi, and has many medical benefits [3,4]. In addition, because of its strong stability and resistance to oxidation at high temperatures, it can find applications in the processing of extended-shelf-life food products, among others [5,6]. These application values make a high oleic acid content an important trait in rapeseed breeding. At present, many rapeseed varieties with a high oleic acid content are bred around the world [7,8]. However, most high-oleic-acid rapeseed has poor resistance and low yield in China [9], which may be related to the mechanisms by which lipid metabolism is regulated, but research on the subject is insufficient.

It has been well established that lipid accumulation in plant seeds is a complex and intertwined biological process [10]. It begins with the synthesis of fatty acids: Acetyl-CoA carboxylase (ACC) catalyzes the first committed step of de novo fatty acid biosynthesis via the carboxylation of acetyl-CoA to malonyl-CoA [11]. Next, Mal-CoA:ACP S-malonyltransferase (MAT) converts malonyl-CoA to malonyl-ACP, which is the primary substrate for a subsequent series of condensation reactions [12]. The fatty acid synthetase system further extends the carbon chain through condensation, reduction, dehydration, and further reduction to obtain various types of fatty acids [13]. These fatty acids and glycerol are used as raw materials to synthesize triacylglycerol (TAG) through the phosphatidic acid pathway [14]. The entire process involves multiple metabolic pathways and many known and unknown genes, which makes the mechanism of oil biosynthesis in rapeseed unclear and hinders the genetic improvement of rapeseed lipids.

Long-chain non-coding RNA (lncRNA) molecules are non-coding transcripts longer than 200 nucleotides, which do not encode proteins and have low conservation [15]. With the rapid development of sequencing technology, lncRNAs have been identified in many plants, such as Arabidopsis [16,17,18], rice [19,20], kiwifruit [21,22], and Chinese cabbage [23], and these lncRNAs play important roles in various stress responses and plant developmental processes. In *Brassica napus*, lncRNAs have been reported to be involved in biological and abiotic stresses, such as antibacterial nuclear disease [24], root swelling disease [25], drought [26], and cadmium toxicity response [27]. In addition, Shen et al. [28] found lncRNAs widely expressed in *Brassica napus* ‘KenC-8′ at two developmental times and found some lncRNAs involved in lipid synthesis. However, compared to these experiments, which were conducted in one place and with only one material, in this study, we conducted the experiment in three different regions, providing further support to their research. To date, the identification of lncRNAs in high-oleic-acid rapeseed using whole transcriptomics has not been reported.

To study the possible role of lncRNAs in regulating lipid metabolism in high-oleic-acid rapeseed, we conduct a comprehensive analysis of lncRNA transcripts at multiple stages of seed development. Next, we used weighted gene correlation network analysis (WGCNA) to screen the key modules of lipid metabolism with the physiological data of the oil content and the fatty acid content. Finally, we constructed an lncRNA–mRNA co-expression network. On a combinatorial basis, our study provides lncRNA–mRNA-based regulatory insights into the genes governing seed development in high-oleic-acid rapeseed.

## 2. Results

### 2.1. Dynamic Changes in the Oil Content and Fatty Acid Components during Seed Development in High- and Low-Oleic-Acid Rapeseed

**Figure 1:**
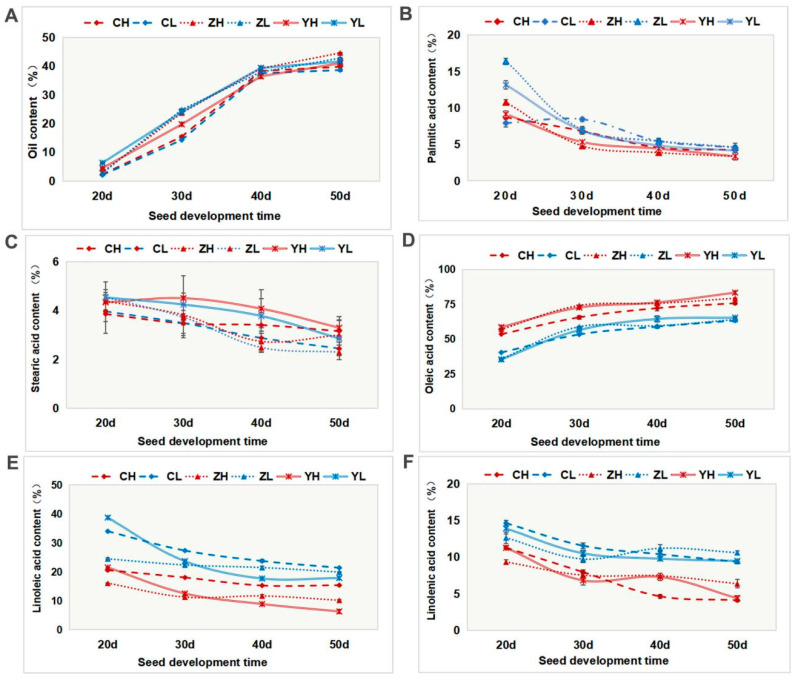
Oil content and fatty acid composition of high- and low-oleic-acid rapeseed seeds at different development times in three regions. (**A**) Oil content, (**B**) palmitic acid content, (**C**) stearic acid content, (**D**) oleic acid content, (**E**) linoleic acid content, and (**F**) linolenic acid content. C, Changsha, Hunan, China; Z, Quzhou, Zhejiang, China; Y, Kunming, Yunnan, China; H, high-oleic-acid rapeseed “Gaoyousuan No.1”; L, low-oleic-acid rapeseed “Xiangyou No.15”; CH and CL, mean high-oleic-acid rapeseed “Gaoyousuan No.1” and low-oleic-acid rapeseed “Xiangyou No.15” planted in Changsha, Hunan, China, respectively; ZH and ZL, high-oleic-acid rapeseed “Gaoyousuan No.1” and low-oleic-acid rapeseed “Xiangyou No.15” planted in Quzhou, Zhejiang, China, respectively; YH and YL, high-oleic-acid rapeseed “Gaoyousuan No.1” and low-oleic-acid rapeseed “Xiangyou No.15” planted in Kunming, Yunnan, China, respectively; 20 d, 30 d, 40 d, and 50 d, different days of seed development (hereinafter the same). Error bars represent the standard error (SE) of three biological replicates.

To identify the lncRNA transcripts related to lipid metabolism, we detected the oil accumulation during the seed development of the “Gaoyousuan No.1 (H)” and “Xiangyou No.15” (L) varieties. We found that the oil content of the two varieties in the three regions shows a trend of increasing from day 20 (20 d) to day 50 (50 d), growing fast at the early stage and slowly at the later stage (Figure 1A). There was no significant difference in the oil content between the two varieties (LSD, *p* > 0.05). The determination of fatty acid composition showed that the change in the oleic acid content was consistent with that of the oil content, both of which showed an increasing trend, and the oleic acid content of H was significantly higher than that of L (LSD, *p* < 0.05). Conversely, the contents of linoleic and linolenic acids in H were always lower than those in L (Figure 1E,F). A correlation analysis showed that the oleic acid content is significantly negatively correlated with the contents of linoleic and linolenic acids (Pearson’s correlation test; *p* < 0.05) and significantly positively correlated with the oil content (Pearson’s correlation test; *p* < 0.05); see Supplementary Table S1.

### 2.2. Whole-Transcriptome Identification of lncRNAs in High- and Low-Oleic-Acid Rapeseed 

We prepared cDNA libraries of polyadenylated RNAs extracted from rapeseed at four different seed developmental stage groups from three regions and generated RNA- seq data sets at a sequencing depth of 84.50 million reads per sample (Supplementary Table S2). Next, the filtered reads were aligned to the “zs11” genome reference sequence using HISAT2 (version 2.1.0, https://ccb.jhu.edu/software/hisat2/index.shtml, accessed on 13 October 2022) [29], and we were able to detect and characterize the expression patterns of ∼90.11% of the known annotated genes (Supplementary Table S3).

**Figure 2:**
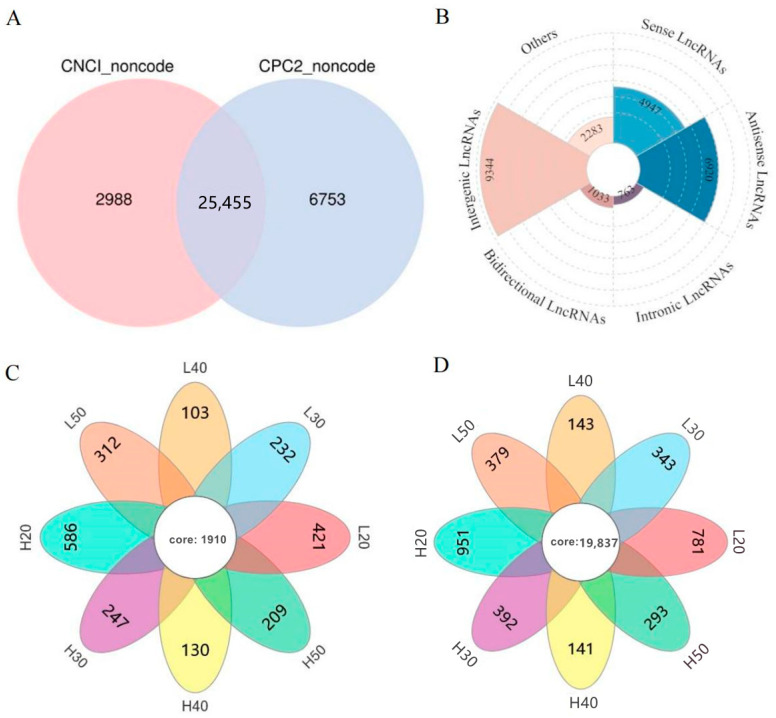
A comprehensive catalogue of lncRNA–mRNA relationships in *Brassica napus* seeds at different development stages. (**A**) Venn diagram of CPC2 and CNCI, (**B**) rose diagram of the lncRNA type, and (**C**,**D**) Venn diagram of the differential expression of lncRNAs and mRNAs in different samples, respectively. H20, H30, H40, and H50, seeds of high-oleic-acid rapeseed “Gaoyousuan No.1” at 20, 30, 40, and 50 days, respectively; L20, L30, L40, and L50, seeds of low-oleic-acid rapeseed “Xiangyou No.15” at 20, 30, 40, and 50 days, respectively.

On the basis of the transcript assembly, 71,333 transcripts (57,287 genes) were reconstructed using stringTie software (version 1.3.4, Johns Hopkins University, Baltimore, MD, USA) [30]. The Coding–Non-Coding Index (CNCI; version 2, https://github.com/www-bioinfo-org/CNCI/, accessed on 14 October 2022) [31] and Coding Potential Calculator 2 (CPC2; version 0.9-r2, http://cpc2.gao-lab.org/, accessed on 14 October 2022) [32] were used to predict the coding ability of the new transcripts. Next, taking the intersection of the transcripts with no coding potential in both software products as possible novel lncRNAs, 25,455 lncRNAs were obtained (Figure 2A). According to the position of each lncRNA in the genome relative to the protein-coding gene, these lncRNAs can be divided into five categories: intergenic lncRNAs, bidirectional lncRNAs, intronic lncRNAs, antisense lncRNAs, and sense overlapping lncRNAs. Intergenic lncRNAs (9344) constitute the largest number (Figure 2B). In addition, we found that compared to the lncRNAs (1068) in L, the number of lncRNAs (1172) specifically expressed in H was slightly larger (Figure 2C). Similarly, there were more mRNAs specifically expressed in H (2717, Figure 2D). Moreover, we also found that the number of mRNAs and lncRNAs specifically expressed in seeds at 20 d was the largest, whether in H or in L (Figure 2C,D). 

### 2.3. Identification of lncRNA and mRNA Expression Patterns

**Figure 3:**
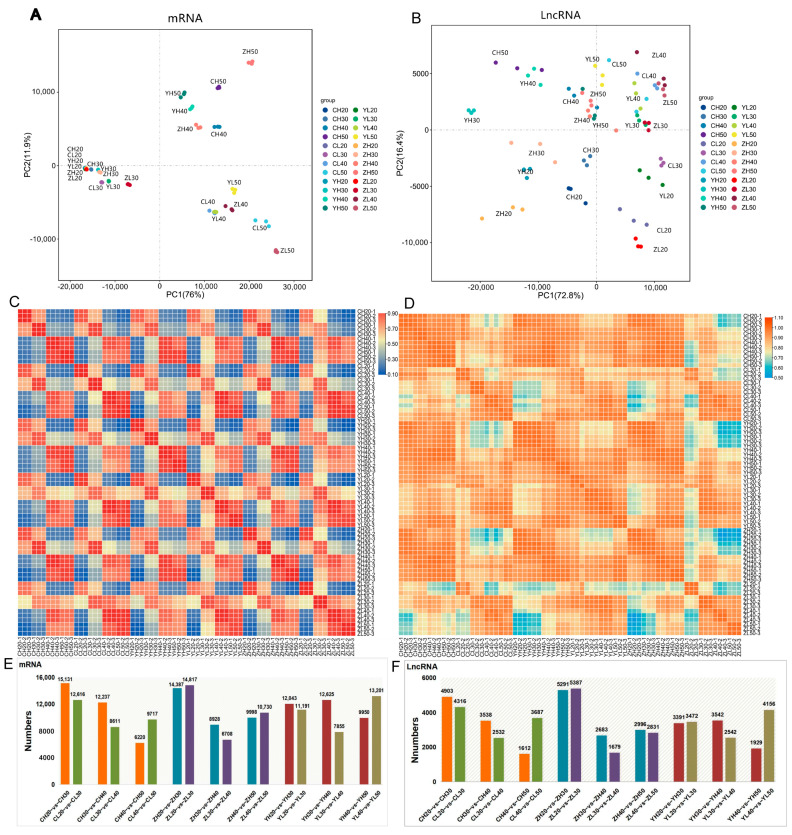
The discrete expression patterns of lncRNAs and mRNAs. (**A**,**B**) Principal component analysis (PCA) of 72 samples from four development stages based on normalized mRNA and lncRNA expression levels, respectively. (**C**,**D**) Heat maps of 72 samples’ correlation coefficients based on mRNA and lncRNA expression levels. (**E**,**F**) Bar graphs of differentially expressed mRNAs (**E**) and lncRNAs (**F**) at adjacent developmental stages. Note: The meaning of sample names is the same as in Figure 1.

To analyze the variation in the expression patterns of all mRNAs and lncRNAs, our data sets were subjected to a principal component analysis (PCA). The mRNA expression pattern at 20 d and 30 d represented a distinct cluster; that in the seeds of high-oleic-acid rapeseed at 40 d and 50 d of development (H40 and H50) represented another and in the seeds of low-oleic rapeseed at 40 d and 50 d of development (L40 and L50) represented a third one (Figure 3A). However, for lncRNA expression, the samples varied greatly in different development times and regions (Figure 3B). A Pearson’s correlation analysis for all pairs of RNA-seq samples was performed, demonstrating similar results (Figure 3C,D). The expression of mRNAs in each cluster was closer than that of lncRNAs, consistent with the higher expression dynamics of lncRNAs. However, we found that the expression level of lncRNAs was lower than that of mRNAs (Supplementary Figure S1).

Next, we determined the differential expression of lncRNAs and mRNAs between any two developmental stage groups (20–30 d, 30–40 d, and 40–50 d) of high- and low-oleic-acid rapeseed. The mRNA of both materials changed greatly from 20 d to 30 d, and the change in H was greater than that in L from 30 d to 40 d (Figure 3E), which is consistent with the principal component analysis (PCA). Such a changing pattern was also evident in the lncRNA expression (Figure 3F).

### 2.4. Discrete Expression Modules of lncRNA and mRNA Expression Using WGCNA Analysis

**Figure 4:**
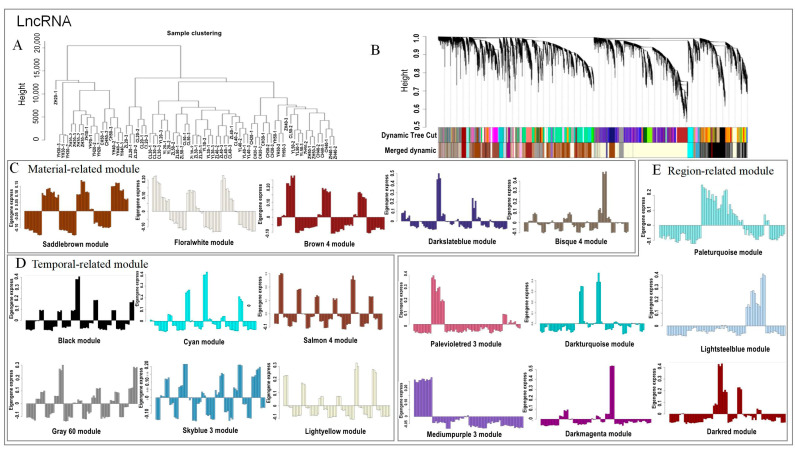
The discrete expression modules of lncRNA expression in WGCNA analysis. (**A**) Sample hierarchical clustering tree, (**B**) module hierarchical clustering diagram, (**C**) material-related modules, (**D**) temporal-related modules, and (**E**) region-related modules. Note: Different colors of (**C**–**E**) represent different expression modes, in the order from left to right and top to bottom, as follows: (**C**) saddlebrown module, floralwhite module, brown 4 module, darkslateblue module, and bisque 4 module; (**D**) black module, cyan module, salmon 4 module, gray 60 module, skyblue 3 module, and lightyellow module; and (**E**) paleturquoise module, palevioletred 3 module, darkturquoise module, lightsteelblue module, mediumpurple 3 module, darkmagenta module, and darkred module.

To characterize the dynamic changes in lncRNA and mRNA expression, we first filtered low-quality genes and obtained 19,440 lncRNAs and 32,632 mRNAs (Supplementary Figure S2). Next, we clustered all their expression patterns using the WGCNA method. We identified 16 major mRNA transcription modules and 21 major lncRNA transcription modules (Figure 4A, Supplementary Figure S3A). Each module had different expression patterns, which are described by the color corresponding to the cluster tree (Figure 4B, Supplementary Figure S3B). This allowed us to define the modules into three classes: material, temporal, and region related (Figure 4C–E, Supplementary Figure S3C–E). The characteristic of the material module is that there is a significant difference in the expression of high- and low-oleic-acid rapeseed types, but the regional regulation is not significant. For example, the saddle-brown module of lncRNAs was downregulated in H and upregulated in L, while the black module of mRNA was, conversely, upregulated in H and downregulated in L (Figure 4C, Supplementary Figure S3C). Similarly, the temporal-related modules are related to different development stages, such as the gray 60 module of lncRNAs, which was highly expressed in the 40 d and 50 d samples, regardless of material and region. The gray 60 module of mRNA was only highly expressed in the 50 d samples (except YH40 samples; Figure 4D, Supplementary Figure S3D). Region-related modules, such as the pale-turquoise module of lncRNAs and the maroon (except ZL20) and light-steel-blue modules (except YH20) of mRNAs, were only highly expressed in the Yunnan samples (Figure 4E, Supplementary Figure S3E).

However, the region-related modules are usually related to the sample or development stages. For example, the pale-violet-red 3 module of lncRNAs was only highly expressed in the Hunan and Yunnan 20–40 d samples, and the dark-turquoise module of mRNAs was only highly expressed in the Yunnan 20–30 d samples. In addition, we found that the difference between the Yunnan sample and samples from the other two regions is large.

### 2.5. Identification of Material- and Temporal-Related lncRNAs and mRNAs

**Figure 5:**
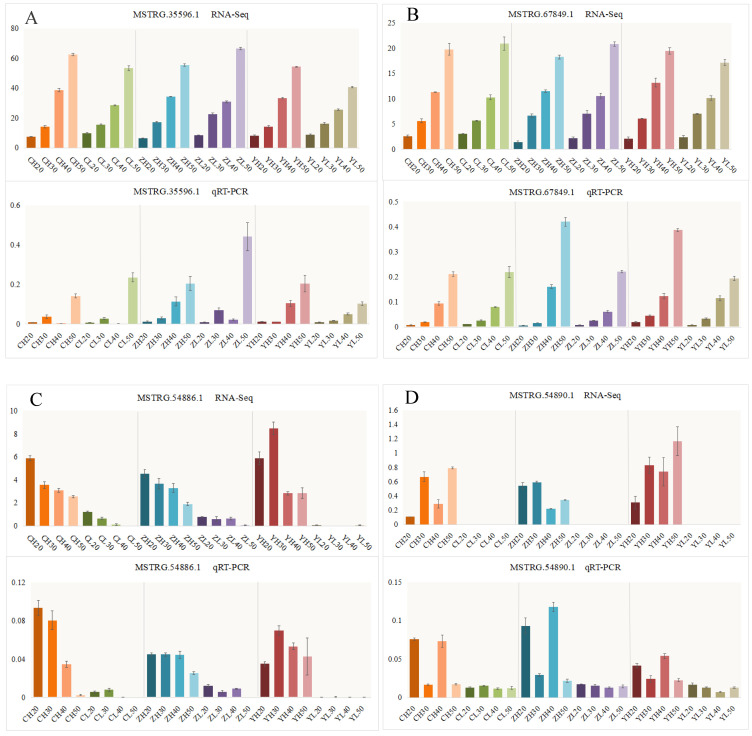
Verification of lncRNAs in the material- and temporal-related modules: (**A**,**B**) lncRNAs in the temporal-related modules and (**C**,**D**) lncRNAs in the material-related modules. Note: The meaning of sample names is the same as in Figure 1. Error bars represent the standard error (SE) of three biological replicates.

Based on the aforementioned analysis, we randomly selected several lncRNAs and mRNAs to verify the differential expression between high- and low-oleic-acid samples in different periods. *MSTRG.35596.1* and *MSTRG.67849.1* belong to the temporal module of lncRNAs, and their expression level increased with the development time (Figure 5A,B). *MSTRG.54886.1* and *MSTRG.54890.1* belong to the material module of lncRNAs, which were only highly expressed in H (Figure 5C,D). Similarly, *BnaA07G0011800ZS* and *BnaC07G0026200ZS* belong to the temporal module of mRNAs (Supplementary Figure S4A,B), and *BnaA02G0049700ZS* and *BnaC02G0057200ZS* belong to the material module of mRNAs (Supplementary Figure S4C,D). The results of qRT-PCR were basically the same as those of RNA-seq, indicating that the sequencing results and WGCNA analysis results are reliable.

### 2.6. Module Analysis Related to the Oil Content and Fatty Acid Combined with Physiological Data

**Figure 6:**
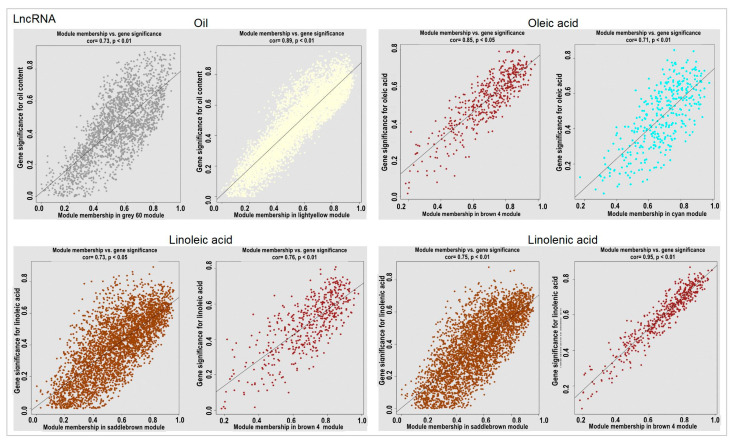
The most positive and negative modules of lncRNAs related to the oil content and fatty acids. Note: For each character, the left side is the most positively correlated module and the right side is the most negatively correlated module.

Combined with physiological data, we explored the lncRNA and mRNA modules related to the oil content, oleic acid, linoleic acid, and linolenic acid (Figure 6; Supplementary Figure S5). The most positive and negative modules related to the oil content in terms of lncRNAs were the gray 60 and light-yellow modules, respectively. The most positive and negative modules related to oleic acid were the brown 4 and cyan modules. The brown 4 module was also the most negative one related to linoleic and linolenic acids, and the most positively related module was the saddle-brown module (Figure 6).

In terms of mRNAs, the most positive and negative modules related to the oil content were the pale-turquoise and light-steel-blue modules, respectively. The most positive and negative modules related to oleic acid were the blue 2 and ivory modules, which were also the most negative and positive modules, respectively, related to linoleic and linolenic acids (Supplementary Figure S5). KEGG analysis showed that these modules are not only enriched in the lipid synthesis and metabolism pathway but also closely related to other life activity pathways (Supplementary Figure S6). 

### 2.7. Construction of lncRNA–mRNA co-Expression Network Related to Lipid Metabolism

**Figure 7:**
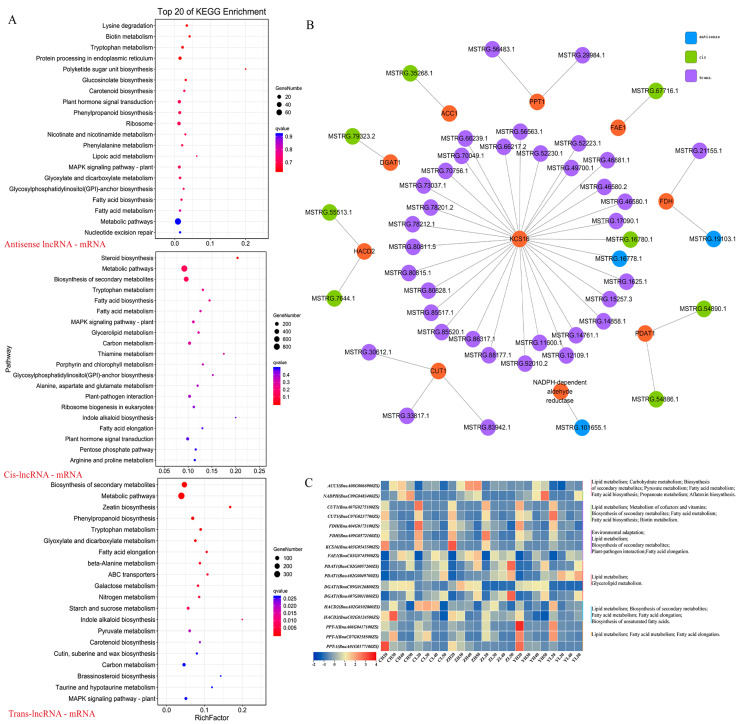
Screening of lncRNAs and mRNAs related to lipid metabolism and co-expression network illustration. (**A**) KEGG pathways for mRNAs associated with antisense, cis-acting, and trans-acting lncRNAs; (**B**) co-expression network related to lipid metabolism; and (**C**) mRNA expression and functional pathways involved in co-expression networks.

Before constructing the lncRNA–mRNA co-expression network, we carried out an association analysis between lncRNAs and mRNAs according to three aspects: base complementary pairing of lncRNAs and mRNAs (antisense analysis), lncRNAs’ regulation of the transcription of their adjacent protein-coding genes (cis-acting analysis), and correlation analysis of lncRNAs and their co-expressed protein-coding genes (trans-acting analysis). The antisense analysis detected 7098 co-expression pairs, which contained 848 differential expression pairs, including 834 lncRNAs and 783 mRNAs (Supplementary Table S3), and the cis-acting analysis detected 68,907 co-expression pairs, which contained 9996 differential expression pairs, including 5744 lncRNAs and 7585 mRNAs (Supplementary Table S3). A total of 33,585 pairs of differential co-expression relationship pairs were detected using trans-effect analysis, including 1501 lncRNAs and 3367 mRNAs (Supplementary Table S3). The KEGG analysis showed that these differential co-expressions are mainly enriched in the metabolism and biosynthesis of amino acids, sugars, fatty acids, and other substances (Figure 7A).

Next, we selected the genes in the fatty acid biosynthesis and metabolism pathway and combined them with the modules screened in Section 2.6. We also screened them under the condition of correlation coefficient ≥ |0.6|, *p* < 0.05. Finally, the lncRNA–mRNA co-expression network related to lipid metabolism was constructed (Figure 7B). It was observed that some genes were not only involved in the biosynthesis and metabolism of fatty acids but also involved in the environmental adaptation and biosynthesis of secondary metabolites. Moreover, these genes were variously expressed in different development stages of high- and low-oleic-acid rapeseed. Taking *3-ketoacyl-CoA synthese 16* (*KCS16, BnaA03G0543500ZS*) and *acyl-CoA:diacylglycerol acyltransferase 1* (*DGAT1, BnaC09G0126800ZS*) as examples, the expression level of both genes in H was higher than that in L, and the expression level of the former decreased with time, while the latter increased with time (Figure 7C). 

However, in this network, one mRNA could be correlated with one or more lncRNAs and one lncRNA could also be associated with one or more mRNAs (Supplementary Table S3). Taking *KCS16*, *fatty acid elongase1* (*FAE1*), and *acetyl-CoA-carboxylase 1* (*ACC1*) genes and their related lncRNAs in the network as examples, we found positive and negative regulatory effects between lncRNAs and mRNAs (Supplementary Figure S7).

## 3. Discussion

### 3.1. Characteristics of lncRNAs: Low Expression Level and High Expression Specificity (in Different Materials, Regions, and Development Stages)

Studies show that compared to protein-coding genes, lncRNAs have a lower expression level [33,34]. Transcripts were filtered in three groups using fpkm values equal or smaller than 5, 10, and 20, and we found that the corresponding mRNAs accounted for 12%, 6%, and 3% of the total mRNAs, while the corresponding lncRNAs accounted for only 4%, 2%, and 1% under the same conditions (Supplementary Table S2). Combined with the violin diagram of the sample expression level (Supplementary Figure S1), we proved, again, that the expression level of most lncRNAs was indeed lower than that of mRNAs. However, Xu et al. [35] compared *Miscanthus lutarioriparius* from two environments, their native habitat neighborhood, and translated fields where the species was introduced by humans. The authors showed that the expression diversity and frequency of lncRNAs in the population are significantly higher than those of protein-encoding mRNAs and that lncRNAs are more sensitive to environmental changes than protein-encoding mRNAs. We also found that the expression specificity of lncRNAs is stronger than that of mRNAs in different materials, regions, and development stages (see Figure 3 and Figure 4, Supplementary Figure S3). For example, PCA showed that the expressed lncRNAs are distributed in different regions and different development stages of high- and low-oleic-acid rapeseed, and WGCNA showed that there are more regional-related lncRNA modules. Among them, the samples from Yunnan are significantly different from those from the other two regions, which might be related to the higher altitude and farther geographical location. It may also be attributed to the generally low expression level of lncRNAs, as well as the limitations in detection using standard mRNA-sequencing protocols [34]. Importantly, this expression specificity of lncRNAs makes them potentially suitable as markers for tissues and developmental stages. 

Our study also showed that oleic acid content has a strong negative correlation with linoleic and linolenic acids and a strong positive correlation with the oil content (Supplementary Table S1). Interestingly, the two mRNA modules that we obtained most positively and negatively correlated with oleic acid are just the most negatively correlated and positively correlated modules of linoleic and linolenic acids (Supplementary Figure S5), while among lncRNAs, only one module, brown 4, has the most positive correlation with oleic acid and the most negative correlation with linoleic and linolenic acids, which indicates that lncRNAs have a higher expression specificity (Figure 6). This difference may be caused by the stronger expression specificity of lncRNAs. For the oil content, although it is positively correlated with oleic acid content, its most positive and negative correlation modules are both different from those of oleic acid. In addition to being possibly related to the specificity of lncRNA expression, Schilbert et al. [36] showed that the oil content is a complex quantitative trait controlled by multiple genes and is susceptible to environmental and other factors, which may be the main reason for its differences. Environmental factors and sample size can be increased for further verification.

### 3.2. The lncRNA–mRNA Relationships in the Core Network Play an Important Role in Regulating Lipid Metabolism

Fatty acids are major components of lipids [37]. From previous studies, we have learned that some genes play important roles in fatty acid regulation. Taking *KCS16* in the core network as an example, *KCS16* is a member of the 3-ketoacyl CoA synthase family involved in very-long-chain fatty acid (VLCFA, C > 20) biosynthesis [38]. This gene is highly expressed in siliques and also substantially in leaves and other organs [39], and the enzyme has no in vitro elongation activity toward C16–C20 substrates [40]. Recent research has also found that Arabidopsis *KCS16* forms C36/C38 acyl precursors for the leaf trichome and pavement surface wax [41].

**Figure 8:**
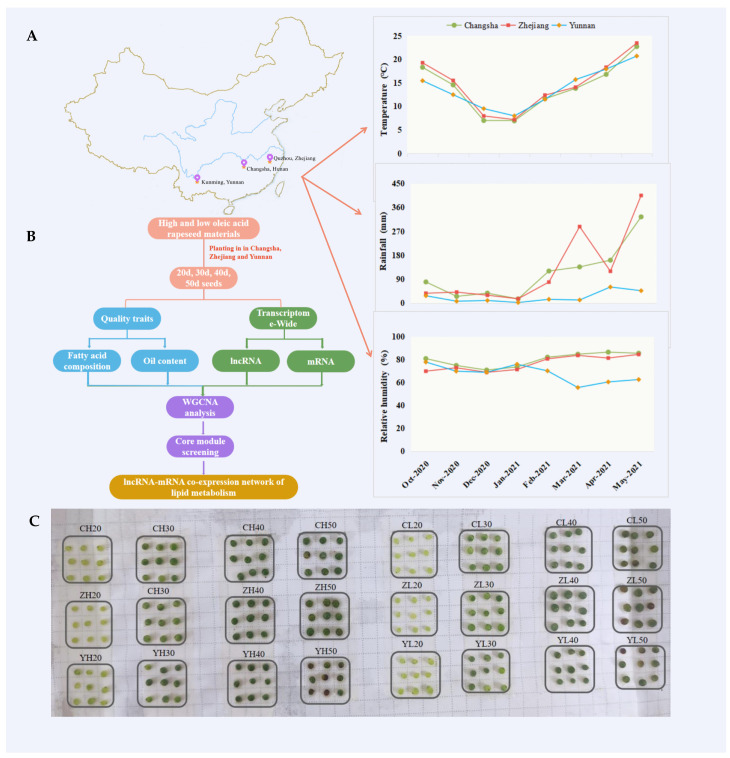
Overview of the research. (**A**) Climate overview of the test sites, (**B**) test technical route, and (**C**) seed map of the four development stages in the three regions. Note: The meaning of sample names in Figure 8C is the same as in Figure 1.

*KCS16*, *FAE1*, *eceriferum* (*CER6*, as known as *CUT1*), and *fiddlehead* (*FDH*) genes belong to the KCS gene family [42]. The FAE1 gene catalyzes the first condensation step of the VLCFA biosynthesis elongation pathway and is the key gene of erucic acid biosynthesis [43,44]. James et al. [45] indicated that *FAE1* is expressed in the developing seed but not in leaves. Ozseyhan et al. [46] found that VLCFAs in the total fatty acids of the camelina fae1 mutant decreases significantly and C18 unsaturated fatty acid increases significantly, while the fae1 mutants have normal seed and plant growth. *CUT1* is required for the elongation of C24 VLCFAs. The inhibition of *CUT1* gene expression leads to severe wax loss and conditional male sterility of transgenic plant stems and pods [47]. The FDH gene is mainly expressed in flower and young leaf organs. Its deletion can increase the permeability of the cell wall and epidermis, lead to organ fusion (such as pollen–stigma fusion), and also change the fatty acid composition [48,49,50]. In addition, our data show that *KCS16*, *CUT1*, and *FDH* decrease with the increase in seed development time (Figure 8C), which is opposite to the change in oleic acid content Figure 1). These genes may influence oleic acid accumulation, but this relationship needs to be studied.

The majority of seed oils are stored in the form of triacylglycerol (TAG) [51]. The two key enzymes that catalyze the last acylation step of TAG production, acyl-CoA:diacylglycerol acyltransferase 1 (DGAT1) and phospholipid:diacylglycerol acyltransferase 1 (PDAT1), are rate-limiting enzymes that determine the TAG accumulation in seeds [52]. A considerable number of studies have shown that *DGAT* affects seed the oil content, TAG content, fatty acid composition, and seed weight [53,54], and *DGAT1* and *PDAT1* have obvious functional redundancy and play important roles in pollen growth and seed development [52,55]. The different copies of these two genes enriched in this experiment had different expression patterns in the samples and may have different regulation patterns, which can be the focus of subsequent studies.

In general, the lipid metabolism mechanism of high-oleic-acid rapeseed is a complex metabolic process regulated by multiple genes in a variety of ways. In addition to these genes, we also obtained their related lncRNAs. How these lncRNAs regulate the corresponding genes to play a role in the lipid metabolism of high-oleic-acid rapeseed is the focus of our future work.

## 4. Material and Methods

### 4.1. Plant Materials, Growth Conditions, and Sample Collection

In this study, *Brassica napus* “Gaoyousuan No.1” (oleic acid content > 75%) and “Xiangyou No.15” (oleic acid content *<* 65%) were used as study materials and were planted in Yunnan (Kunming, 102.72° E, 25.04° N), Zhejiang (Quzhou, 118.87° E, 28.97° N), and Hunan (Changsha, 113.03° E, 28.18° N) from October 2020 to May 2021. The climatic conditions of the three regions are shown in Figure 8A. Each material was planted in a plot with an area of 2 × 4 = 8 m^2^ and a density of 150,000 plants hm^−2^. Three biological replicates were set.

The plants were marked on the day of flowering, and the seeds of 10 plants were harvested and mixed well on the 20th, 30th, 40th, and 50th days (Figure 8C). The seeds were divided into two parts: one part was snap-frozen in liquid nitrogen and stored at −80 °C for RNA extraction, and the other part was blanched at 120 °C for 30 min and dried at 60 °C to constant weight for the analysis of the oil content and fatty acid composition.

### 4.2. Seed Oil Extraction and Fatty Acid Composition Analysis

The seed oil was extracted with petroleum ether through Soxhlet extraction (GB/T2906-1982) [56]. The specific method is as follows: First, each seed sample was pulverized, weighing 1–2 g, and wrapped in weighed dry filter paper (the filter paper weight was recorded as W0); after ensuring that they did not leak out, we weighed them as W1. Next, the wrapped samples were placed into the Soxhlet extraction device and heated at 60 °C in a water bath, and the oil was extracted with petroleum ether. The samples were removed from the device until the extract was completely colorless. Finally, we waited until the residual petroleum ether was completely volatilized, weighed the samples again (sample and paper), and recorded the weight as W2. The formula for calculating the oil content is (%) = (W2 − W0)/(W1 − W0) × 100%. The measurements were repeated three times for each sample, after which the average value was taken. The least significant difference (LSD) method was used for multiple comparisons.

The fatty acid composition was separated and identified using gas chromatography (Agilent, Santa Clara, CA, USA) [57]. The specific method is as follows: The pulverized seeds were extracted with 800μL ether:petroleum ether (1:1) and esterified with a 400 uL KOH–methanol solution (0.4 mol·L^−1^), followed immediately by shaking and standing. After 4 h of standing, the samples were mixed with distilled water and centrifuged. The organic phase containing fatty acid methyl esters (100 μL) was collected and diluted with petroleum ether (500 μL) and analyzed using a gas chromatograph (Agilent 7890B, USA). Mixed external standards of fatty acids (Sigma, Saint Louis, MO, USA) were used to identify the fatty acids. The identities of all peaks in the chromatograms were determined by comparing their retention times with those of standard fatty acid methyl esters. The fatty acid compositions were expressed as percentages of the sum of the peak areas.

Statistical analysis was performed using SPSS24.0 software (SPSS, IBM, Armonk, NY, USA). The least significant difference (LSD) method was used for multiple comparisons. Pearson’s correlation test was used to assess correlation. Related graphs were generated using Origin2018 software (OriginLab, Northampton, MA, USA).

### 4.3. RNA Extraction, Strand-Specific Library Construction, and Illumina Sequencing 

Total RNA was extracted using the Trizol reagent kit (Invitrogen, Carlsbad, CA, USA) according to the manufacturer’s protocol. RNA quality was assessed on an Agilent 2100 Bioanalyzer (Agilent Technologies, Palo Alto, CA, USA) and checked using RNase-free agarose gel electrophoresis. After the total RNA was extracted, the rRNAs were removed to retain the mRNAs and ncRNAs. The enriched mRNAs and ncRNAs were fragmented into short fragments using fragmentation buffer and reverse-transcribed into cDNA with random primers. Second-strand cDNA were synthesized using DNA polymerase I, RNase H, dNTP (dUTP instead of dTTP), and a buffer. Next, the cDNA fragments were purified with the Qia Quick PCR extraction kit (Qiagen, Venlo, the Netherlands), end-repaired, mixed with poly(A), and ligated to Illumina sequencing adapters. Further, uracil-N-glycosylase (UNG) was used to digest the second-strand cDNA. The digested products were size-selected using agarose gel electrophoresis, PCR-amplified, and sequenced using Illumina HiSeqTM 4000 (Gene Denovo Biotechnology Co., Guangzhou, China).

### 4.4. Sequence Data Analysis

Raw reads obtained from the sequencing machines were filtered using fastp (version 0.18.0) [58] to obtain clean reads. The short-read alignment tool Bowtie2 (version 2.2.8) [59] was used for mapping the clean reads to the ribosomal RNA (rRNA) database. The rRNA-mapped reads were then removed. The remaining reads were mapped to the reference genome using HISAT2 (version 2.1.0, https://ccb.jhu.edu/software/hisat2/index.shtml, accessed on 13 October 2022) [29]. Next, the reconstruction of the transcripts was conducted using Stringtie software (version 1.3.4, Johns Hopkins University, Baltimore, MD, USA) [30]. After reconstruction, on the one hand, we found out genes in the sequencing results but not included in the reference genome and defined them as new genes. On the other hand, transcripts with exon number > 1 and exon length ≥ 200 nucleotides (nt) were selected, the Coding–Non-Coding Index (CNCI; version 2, https://github.com/www-bioinfo-org/CNCI/, accessed on 14 October 2022) [31] and Coding Potential Calculator 2 (CPC2; version 0.9-r2, http://cpc2.gao-lab.org/, accessed on 14 October 2022) [32] software were used to predict the coding potential of transcripts, and the common transcripts without coding potential were selected as reliable new lncRNAs. The lncRNAs were classified according to their location, and a relationship analysis of the samples, using principal component analysis (PCA) and correlation analysis, was performed with the R package gmodels (http://www.rproject.org/). mRNA and lncRNA differential expression analysis was performed using DESeq2 software (v1.25.9) [60] between two different groups (and using edgeR [61] between two samples). Genes/transcripts with a false discovery rate (FDR) below 0.05 and absolute fold-change ≥ 2 were considered differentially expressed genes/transcripts. 

### 4.5. WGCNA Analysis

Before WGCNA, we removed the low-quality genes or samples that had an unstable impact on the results. Next, according to the expression of all genes, all sample processes were clustered hierarchically. The gene expression values were imported into the WGCNA (v1.47) package in R [62] to construct co-expression modules using the automatic network construction function blockwiseModules with default settings, except that the power was 6, TOMType was unsigned, mergeCutHeight was 0.15, and minModuleSize was 50. Correlation analysis was performed using the module eigengene with data for the oil content and fatty acid composition and specific traits. Pearson’s correlation (the *p*-value was calculated using Student’s *t*-test) between each gene and trait data under the module was also calculated for the most relevant module (positive and negative correlations) corresponding to each phenotype data, and the gene significance (GS) value was obtained. For genes in each module, GO and KEGG pathway enrichment analyses were conducted to analyze the biological functions of modules. The lncRNA–mRNA co-expression network was visualized using Cytoscape_3.3.0 [63]. 

### 4.6. qRT-PCR Analysis

Primers were designed using Premier 6.0 and synthesized by Sangon Biotech Co., Ltd. (Wuhan, China). The RNA in Section 4.3 was reverse-transcribed into cDNA following the TransScript One Step gDNA Removal and cDNA Synthesis SuperMix instructions. After the PCR detection of the primer, according to the instructions of the TransScript Tip Green qRT-PCR Super Mix, a two-step qRT-PCR test was conducted using the CFX96 TM Real Time System (BIORAD, USA). Actin was used as an internal reference gene [64], and the method by Pfaffl et al. [65] was used to evaluate gene expression. The primer sequences of randomly selected mRNA and lncRNA are shown in Supplementary Table S4. The experiment was repeated three times, and the average value was taken.

## 5. Conclusions

In summary, we screened and identified lncRNAs and mRNAs at four different developmental stages of high- and low-oleic-acid rapeseed in three regions. Next, 21 lncRNA and 14 mRNA modules were identified using WGCNA, and the modules most related to the oil content, oleic acid, linoleic acid, and linolenic acid were obtained. Finally, a lipid-metabolism-related network was constructed. Our results provide fresh insight into region-, development-stage-, and material-biased changes in lncRNA expression in the seeds of *Brassica napus*. Our network discovered some lncRNAs related to lipid metabolism genes. The manner in which these lncRNAs regulate target genes will be the focus of our subsequent work.

## Data Availability

All the data included in this study are available upon request by contacting the corresponding author.

## Supplementary Materials

The following supporting information can be downloaded at https://www.mdpi.com/article/10.3390/ijms24076277/s1.
