# Supplementary material for: Integrated Analysis of lncRNA–mRNA Regulatory Networks Related to Lipid Metabolism in High-Oleic-Acid Rapeseed
Source: Int J Mol Sci. 2023 Mar 27;24(7):6277. doi: 10.3390/ijms24076277 (PMC10093948; doi:10.3390/ijms24076277)
Supplement: Supplementary file 1 [file ijms-24-06277-s001.zip › Supplementary Figure S1.pdf]

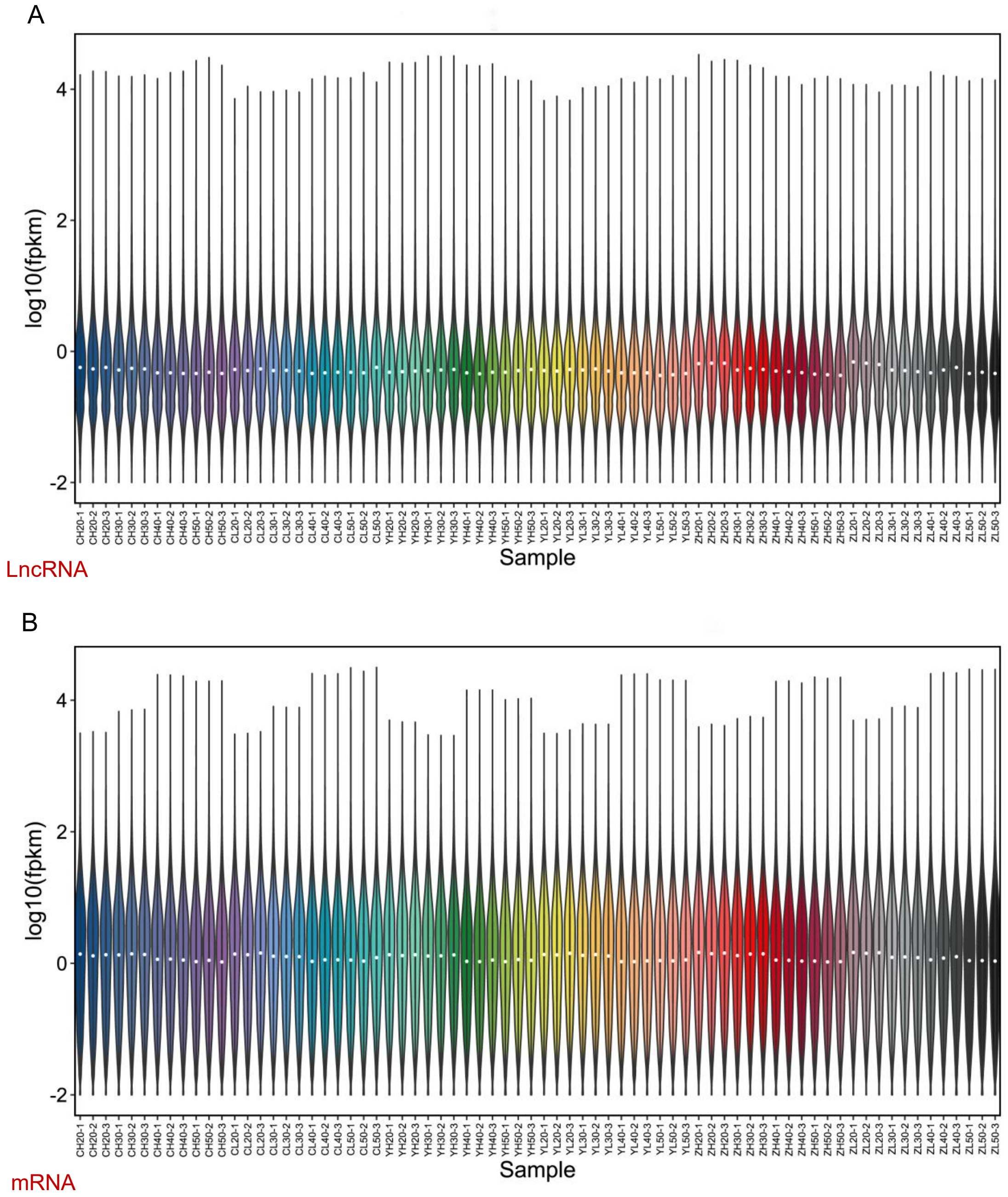

Supplementary Figure S1 Violin plots of the expression of several lncRNAs and mRNAs in the samples under study. (A) violin plots of the expression of several lncRNAs and (B) violin plots of the expression of several mRNAs.
