# Supplementary material for: Integrated Analysis of lncRNA–mRNA Regulatory Networks Related to Lipid Metabolism in High-Oleic-Acid Rapeseed
Source: Int J Mol Sci. 2023 Mar 27;24(7):6277. doi: 10.3390/ijms24076277 (PMC10093948; doi:10.3390/ijms24076277)
Supplement: Supplementary file 1 [file ijms-24-06277-s001.zip › Supplementary Figure S3.pdf]

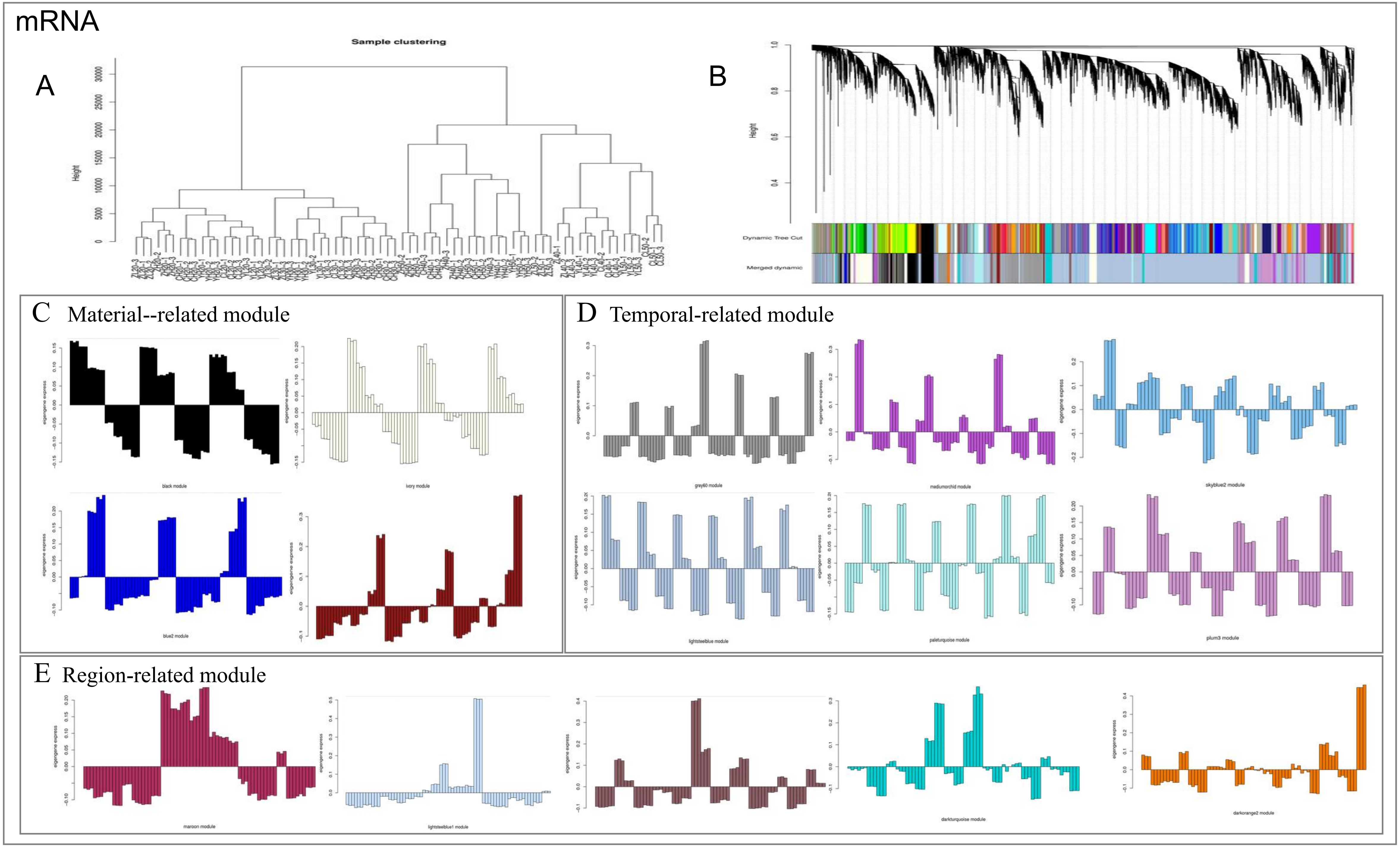

Figure S3. The discrete expression modules of lncRNA expression by WGCNA analysis. (A) Sample hierarchical clustering tree; (B) module hierarchical clustering diagram; (C) material-related modules; (D) temporal-related modules; and (E) region-related modules. Note: Different colors of (C)–(E) represent different expression modules, in the order from left to right and top to bottom, as follows: (C): black module, ivory module, blue2 module, and firebrick4 module; (D): grey60 module, mediumorch module, skyblue2 module, lightsteelblue module, paleturquoise module, plum3 module; (E): maroon module, lightsteelblue1 module, lightpink4 module, darkturquoise module, darkorange2 module
