# Supplementary material for: Integrated Analysis of lncRNA–mRNA Regulatory Networks Related to Lipid Metabolism in High-Oleic-Acid Rapeseed
Source: Int J Mol Sci. 2023 Mar 27;24(7):6277. doi: 10.3390/ijms24076277 (PMC10093948; doi:10.3390/ijms24076277)
Supplement: Supplementary file 1 [file ijms-24-06277-s001.zip › Supplementary Figure S4.pdf]

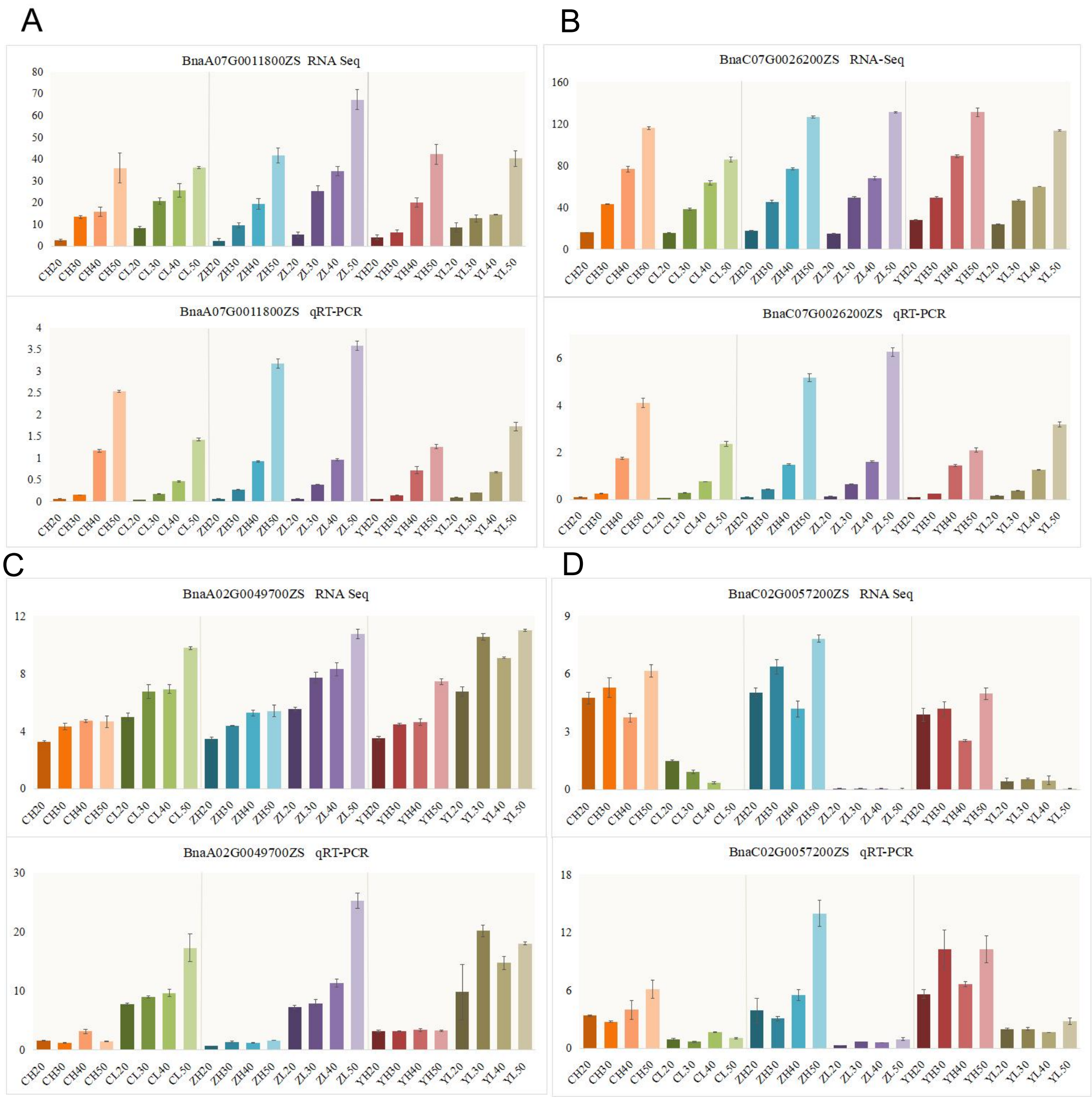

Supplementary Figure S4 Verification of mRNAs in the material- and temporal-related modules.(A) and (B) mRNA in temparal-related modules; (C) and (D) mRNA in material-related modules
