# Supplementary material for: Integrated Analysis of lncRNA–mRNA Regulatory Networks Related to Lipid Metabolism in High-Oleic-Acid Rapeseed
Source: Int J Mol Sci. 2023 Mar 27;24(7):6277. doi: 10.3390/ijms24076277 (PMC10093948; doi:10.3390/ijms24076277)
Supplement: Supplementary file 1 [file ijms-24-06277-s001.zip › Supplementary Figure S6.pdf]

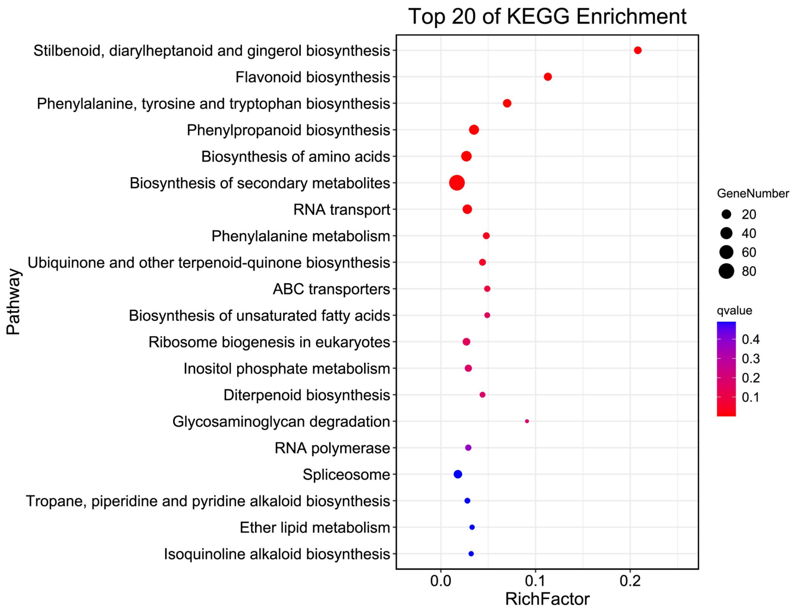

Oil content-paleturquoise model KEGG

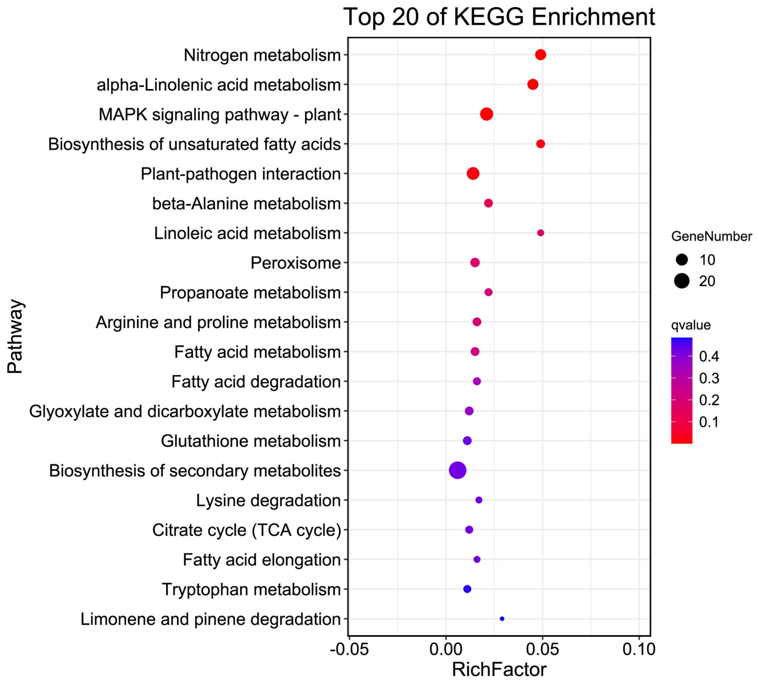

Oil content-lightsteelblue1 model KEGG

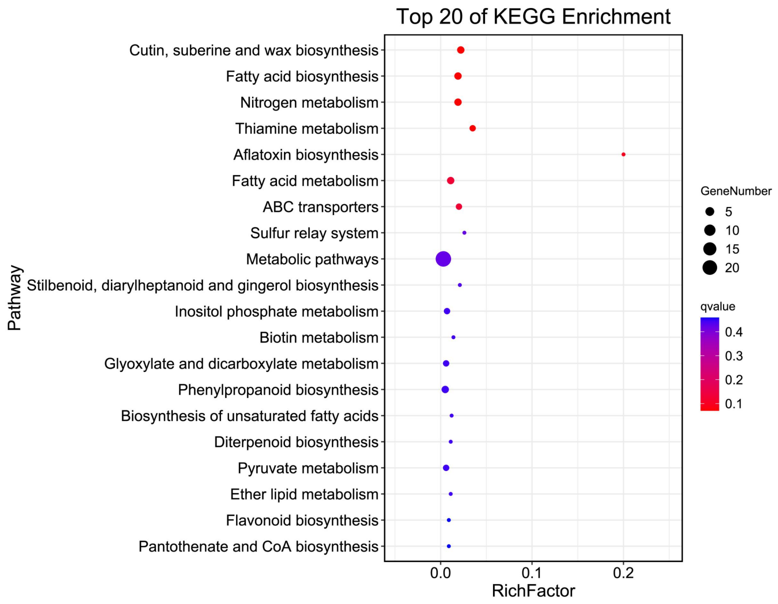

oleic acid, linoleic acid and linolenic acid- blue2 model KEGG

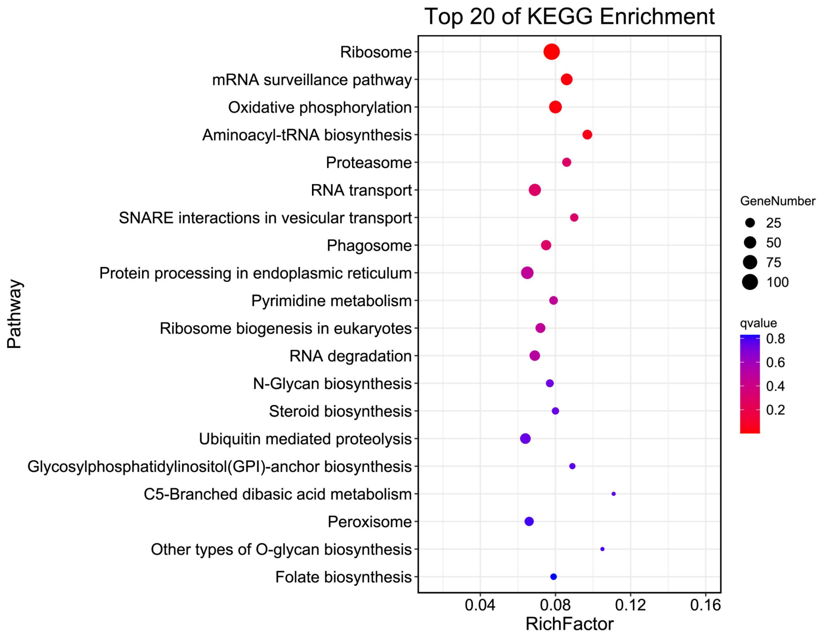

oleic acid, linoleic acid and linolenic acid- ivory model KEGG

Supplementary Figure S6 KEGG analysis of lncRNA and mRNA modules related to oil content, oleic acid, linoleic acid and linolenic acid
