# Supplementary material for: Integrated Analysis of lncRNA–mRNA Regulatory Networks Related to Lipid Metabolism in High-Oleic-Acid Rapeseed
Source: Int J Mol Sci. 2023 Mar 27;24(7):6277. doi: 10.3390/ijms24076277 (PMC10093948; doi:10.3390/ijms24076277)
Supplement: Supplementary file 1 [file ijms-24-06277-s001.zip › Supplementary Table S1. Correlation analysis betwee.pdf]

Supplementary Table S1 Correlation analysis between oil content and fatty acid composition

	oleic acid	linoleic acid	linolenic acid	palmitic acid	stearic acid	oil content
oleic acid	1					
linoleic acid	-0.911**	1				
linolenic acid	-0.910**	0.861**	1			
palmitic acid	-0.857**	0.635**	0.667**	1		
stearic acid	-0.424*	0.226	0.261	0.653**	1	
oil content	0.728**	-0.546**	-0.608**	-0.834**	-0.753**	1

Note：the correlation based on Pearson correlation test.

* Correlation is significant at the 0.05 level (two-tailed); ** correlation is significant at the 0.01 level (two-tailed).
