# Supplementary material for: Integrated Analysis of lncRNA–mRNA Regulatory Networks Related to Lipid Metabolism in High-Oleic-Acid Rapeseed
Source: Int J Mol Sci. 2023 Mar 27;24(7):6277. doi: 10.3390/ijms24076277 (PMC10093948; doi:10.3390/ijms24076277)
Supplement: Supplementary file 1 [file ijms-24-06277-s001.zip › Supplementary Table S4.pdf]

Supplementary Table S4 Primer information of qRT-PCR

Primer name	Primer sequence（5' - 3'）
M35596-F	GATCGTTTTTCTGGCCGACG
M35596-R	AAAAGCGACAGACCACCGAT
M67849-F	CACTCATCGGCACTCAAGGT
M67849-R	CACTCATCGGCACTCAAGGT
M54886-F	GGCTCCTCTGCTGGGTTTATT
M54886-R	ACCACAGCATCCACATGGTA
M54890-F	CAGAAGCAACTGCCATTCGG
M54890-R	AGTTTCAGAAAGCAAGGCCA
BnaA07G0011800ZS-F	CCTCCAACGGACTTCTTCCTGATTC
BnaA07G0011800ZS-R	GCCTCCACCTGATTCCCTAATTTCG
BnaC07G0026200ZS-F	TTCCGTAACTGTTTCCGATGCTGAC
BnaC07G0026200ZS-R	GACTCTCCCTCACCCTCCGATG
BnaA02G0049700ZS-F	TGGATTCGTGCTGCTGGTTCATC
BnaA02G0049700ZS-R	GGATGCTTCGCCTTGAGACCTTC
BnaC02G0057200ZS-F	TGGATTCGTGCTGCTGGTTCATAG
BnaC02G0057200ZS-R	GCTTCGCCCTGAGACCTTCTTTC
Act-F	GGTTGGGATGGACCAGAAGG
Act-R	TCAGGAGCAATACGGAGC
