# Supplementary figures and images for: Integrated Analysis of lncRNA–mRNA Regulatory Networks Related to Lipid Metabolism in High-Oleic-Acid Rapeseed
Source: Int J Mol Sci. 2023 Mar 27;24(7):6277. doi: 10.3390/ijms24076277 (PMC10093948; doi:10.3390/ijms24076277)

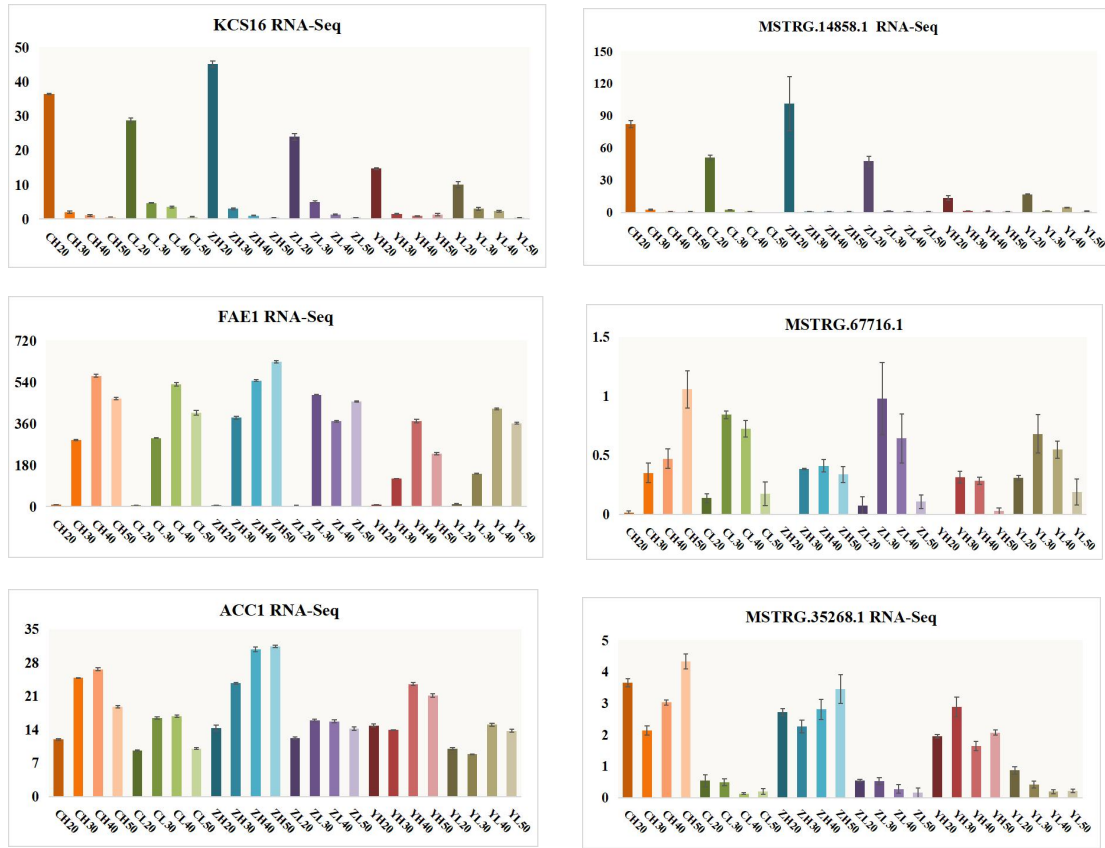

Supplementary Figure S7 The lncRNA and mRNA expression in the co-expression network

Supplement: Supplementary file 1 [file ijms-24-06277-s001.zip › Supplementary Figure S7.pdf]
